# Whole genome prediction for preimplantation genetic diagnosis

**DOI:** 10.1186/s13073-015-0160-4

**Published:** 2015-04-08

**Authors:** Akash Kumar, Allison Ryan, Jacob O Kitzman, Nina Wemmer, Matthew W Snyder, Styrmir Sigurjonsson, Choli Lee, Milena Banjevic, Paul W Zarutskie, Alexandra P Lewis, Jay Shendure, Matthew Rabinowitz

**Affiliations:** Department of Genome Sciences, University of Washington School of Medicine, Seattle, WA 98195 USA; Natera Inc, San Carlos, CA 94070 USA; Department of Obstetrics and Gynecology, University of Washington School of Medicine, Seattle, WA 98195 USA

## Abstract

**Background:**

Preimplantation genetic diagnosis (PGD) enables profiling of embryos for genetic disorders prior to implantation. The majority of PGD testing is restricted in the scope of variants assayed or by the availability of extended family members. While recent advances in single cell sequencing show promise, they remain limited by bias in DNA amplification and the rapid turnaround time (<36 h) required for fresh embryo transfer. Here, we describe and validate a method for inferring the inherited whole genome sequence of an embryo for preimplantation genetic diagnosis (PGD).

**Methods:**

We combine haplotype-resolved, parental genome sequencing with rapid embryo genotyping to predict the whole genome sequence of a day-5 human embryo in a couple at risk of transmitting alpha-thalassemia.

**Results:**

Inheritance was predicted at approximately 3 million paternally and/or maternally heterozygous sites with greater than 99% accuracy. Furthermore, we successfully phase and predict the transmission of an *HBA1/HBA2* deletion from each parent.

**Conclusions:**

Our results suggest that preimplantation whole genome prediction may facilitate the comprehensive diagnosis of diseases with a known genetic basis in embryos.

**Electronic supplementary material:**

The online version of this article (doi:10.1186/s13073-015-0160-4) contains supplementary material, which is available to authorized users.

## Background

Preimplantation genetic diagnosis (PGD) enables profiling of embryos for genetic disorders prior to implantation. Multiple embryos created by *in vitro* fertilization (IVF) are biopsied and screened for aneuploidy and/or single-gene mutations, followed by selective implantation of euploid embryos lacking the targeted disease alleles. A variety of approaches have been developed using different DNA sources (polar body, blastomere, trophectoderm), and different molecular techniques (for example, PCR, FISH, SNP array, or arrayCGH) [1-6]. Although it is in widespread clinical use, current practices for PGD are generally directed at specific loci and/or at chromosomal aberrations [1,7].

In principle, it should be possible to determine the entire genome sequence directly from an embryo, for instance to predict the inheritance of risk alleles for Mendelian disorders or complex, multifactorial phenotypes. However, progress towards this goal is challenged by several practical considerations. First, stochastic biases during the amplification of minute quantities of genomic DNA obtained from embryo biopsies (1 to 10 cells each) both limit and obscure the ascertainment of inherited alleles, while also introducing artifactual mutations [8-10]. Second, single-cell amplification and whole genome sequencing workflows still exceed the 1- to 5-day turnaround time necessary for fresh embryo transfer. Although there have been recent important advances with respect to both low-input amplification protocols and the speed of DNA sequencing [11-14], existing methods are either unable to predict the whole genome sequences of embryos or will necessitate extensive changes in the handling/preparation of embryo biopsies to do so.

Here we show that the combination of rapid embryo genotyping and molecular phasing of parental genomes can be used to predict the whole genome sequence of an embryo within a timeframe suitable for fresh transfer (or alternatively with frozen transfer). This work builds on recently developed methods to experimentally determine an individual’s haplotypes on a genome-wide scale [14-18]. We begin by using genotypes from parents and sibling embryos to accurately predict a sparse subset of genotypes from single or few-cell embryo biopsies despite substantial allele dropout observed in single-cell amplification [19]. We then combine this information with haplotype-resolved genome sequencing of both parents to predict the inherited genome sequence of the embryo.

## Methods

### DNA isolation, whole-genome shotgun library preparation, sequencing, and variant calling

DNA from subjects was obtained after written informed consent. This study was approved by the E&I West Coast Board Institutional Review Board and conducted in accordance with the Declaration of Helsinki. Genomic DNA was extracted from whole blood or saliva samples, as available. Purified DNA was fragmented by sonication with the Covaris S2 instrument. Shotgun sequencing libraries were prepared by either the KAPA library preparation kit (Kapa Biosystems) or ThruPLEX-FD (Rubicon Genomics) following manufacturer’s instructions. All libraries were sequenced on HiSeq 2000 instruments (Illumina) using paired-end 100-bp reads. Reads were mapped to the human reference genome sequence (hg19) with bwa v0.7.1 [20]. After removal of PCR duplicate pairs using the Picard toolkit, local realignment around indels, variant discovery, and quality score recalibration were performed with the Genome Analysis Toolkit (GATK) [21] using standard procedures with an additional filter (HRun <5) to remove potentially spurious variants near mononucleotide repeats [21].

### Dilution pool construction, sequencing, and haplotype phasing

Haplotype-resolved genome sequencing was performed for each parent using a modified Long Fragment Read protocol [11] involving limiting dilution of DNA into 96-well plates followed by amplification, library preparation, sequencing, and alignment (Additional file 1: Note S1). Fragment boundaries were determined as previously described [16] and basecalls were made at heterozygous sites previously determined by whole genome sequencing. Overlapping fragments were assembled into haplotype blocks using the RefHap haplotype assembly package [22] (Additional file 1: Note S2). These ‘molecular haplotypes’ were subsequently expanded by local statistical phasing using haplotypes from the 1000 Genomes project (Additional file 1: Note S3).

### Copy number estimates from whole genome sequencing

Read depths from bwa alignments were aggregated in 100-bp windows and divided by the mean autosomal read depth to correct for differing amounts of coverage between samples. Each library was individually corrected using a smoothed linear model of read depths to G + C composition at each window, as previously described in Sudmant *et al.* [23].

### Embryo genotyping and ‘Parental Support’ analysis

Genotyping was performed on trophectoderm biopsies (day-5, n = 10). Embryo biopsies were subjected to DNA extraction, amplification and genotyping with parents and grandparents using a rapid microarray protocol as previously described in Johnson *et al.* [6] with the Illumina CytoSNP-12 chip used across all samples. Sibling embryo and parent SNP array measurements were combined to improve accuracy of embryo genotypes in the ‘Parental Support’ (PS) method [24] (Additional file 1: Figures S1 and S2). First, a statistical model was employed to determine the Maximum Likelihood Estimate (MLE) phase of heterozygous SNVs in each parent by combining recombination frequencies from the HapMap database [25] with SNP array measurements from parents and SNP array measurements from sibling embryos (Additional file 1: Note S4). Second, PS embryo genotypes were determined using an HMM that finds the most likely parental haplotype transmitted to each embryo given SNP array measurements from the embryo and MLE phase for each parent (Additional file 1: Note S5).

### Whole genome prediction of inherited variants

The whole genome sequence of the embryo was predicted by combining PS embryo genotypes with parental haplotype blocks (Additional file 1: Figures S3, S4, and S5). For each haplotype block, which can be assumed to have two haplotypes A or B, transmission was determined based on the intersection of sites contained in each block and sites called by PS embryo genotypes. The haplotype block (A or B) with the highest score was considered to be transmitted. In the event that a block appeared to be partially transmitted, the block was split conservatively with new boundaries defined by the location of the nearest two embryo genotypes (Additional file 1: Figure S5). Transmission of each of these sub-blocks was determined independently. This process was continued across the entire genome for both the mother and father (Additional file 1: Figure S3). To predict transmission of heterozygous deletions, we manually selected high-quality haplotype blocks from dilution pool sequencing that overlapped the deletion of interest and used other heterozygous SNVs within the haplotype block to determine whether the deletion was transmitted to the embryo.

### Incorporating grandparental information into analysis

Parental haplotypes were obtained using GATK PhaseByTransmission with variants from shotgun sequencing of parents and grandparents used as inputs. Phased genotypes were subsequently incorporated into parental haplotype blocks and PS embryo genotypes (Additional file 1: Note S6). The embryo genome was predicted as previously described both with and without grandparental genotypes and the union of calls was used to determine the increase in prediction coverage with grandparents. Additionally, the embryo genome was predicted with just the grandparental information as well as with only the grandparental genotypes plus the population phased estimates as described in Additional file 1: Note S3. Parental variants were annotated using Variant Effect Predictor [26].

## Results

**Figure 1:**
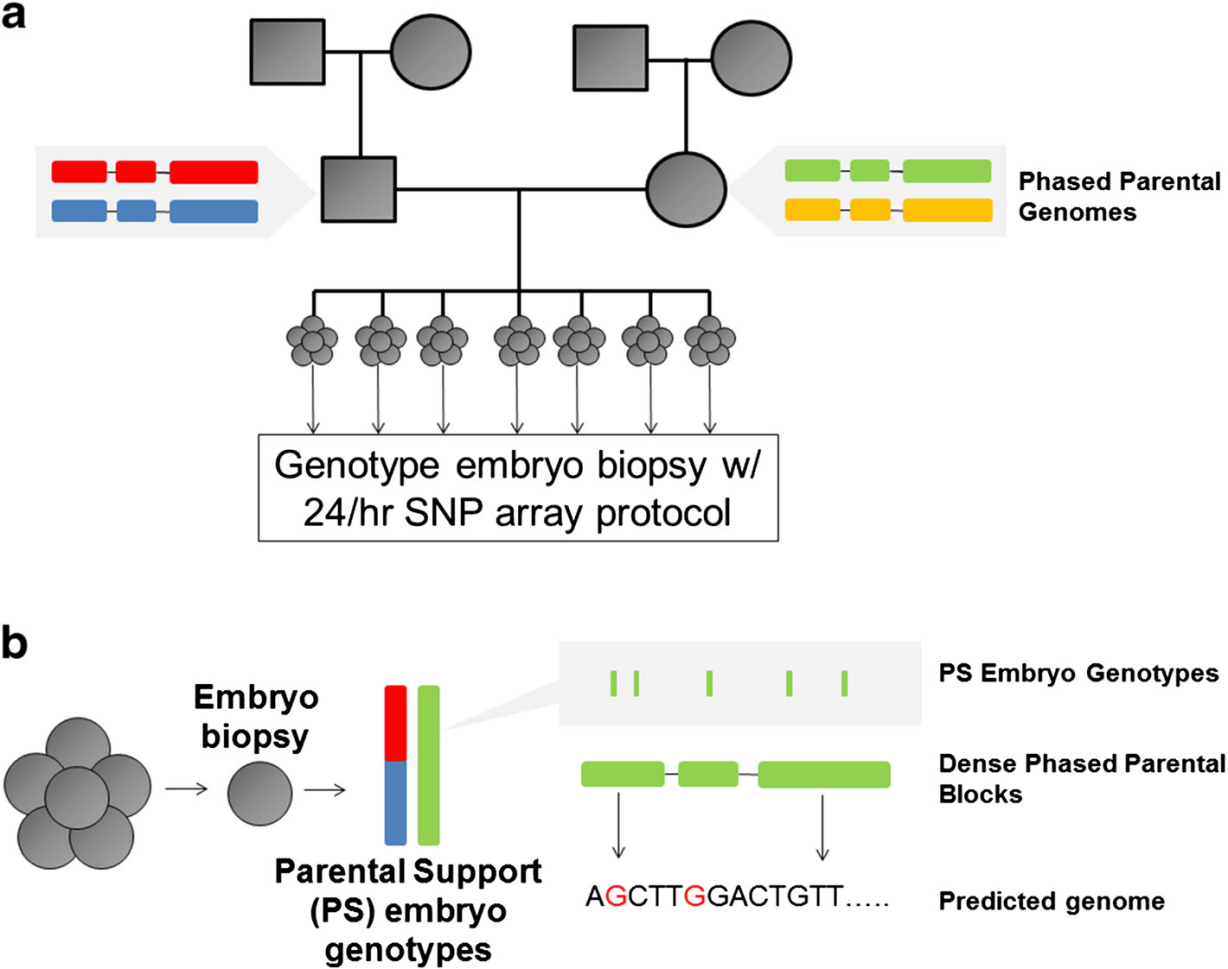
Experimental approach. **(a)** Parental genomes were haplotype-resolved using a combination of direct molecular methods and population-based phasing. Embryo biopsies were genotyped using a rapid SNP-array protocol and processed using information derived from parents and sibling embryos to infer a sparse set of ‘Parental Support’ (PS) genotypes. **(b)** Prediction of the embryo genome. Informative sites from PS embryo genotyping were combined with haplotype-resolved parental genome sequences to predict the whole genome sequence of the embryo.

Materials used in this study were retrospectively obtained from a couple at risk of transmitting alpha-thalassemia that previously underwent a successful round of IVF with preimplantation genetic diagnosis (Additional file 1: Table S1). We obtained trophectoderm biopsies from a total of 10 embryos (day 5) and genotyped each across a panel of 300,000 common SNPs using an expedited, 24-h microarray protocol [6]. Additionally, we genotyped each parent and all four grandparents across the same panel (Figure 1).

**Figure 2:**
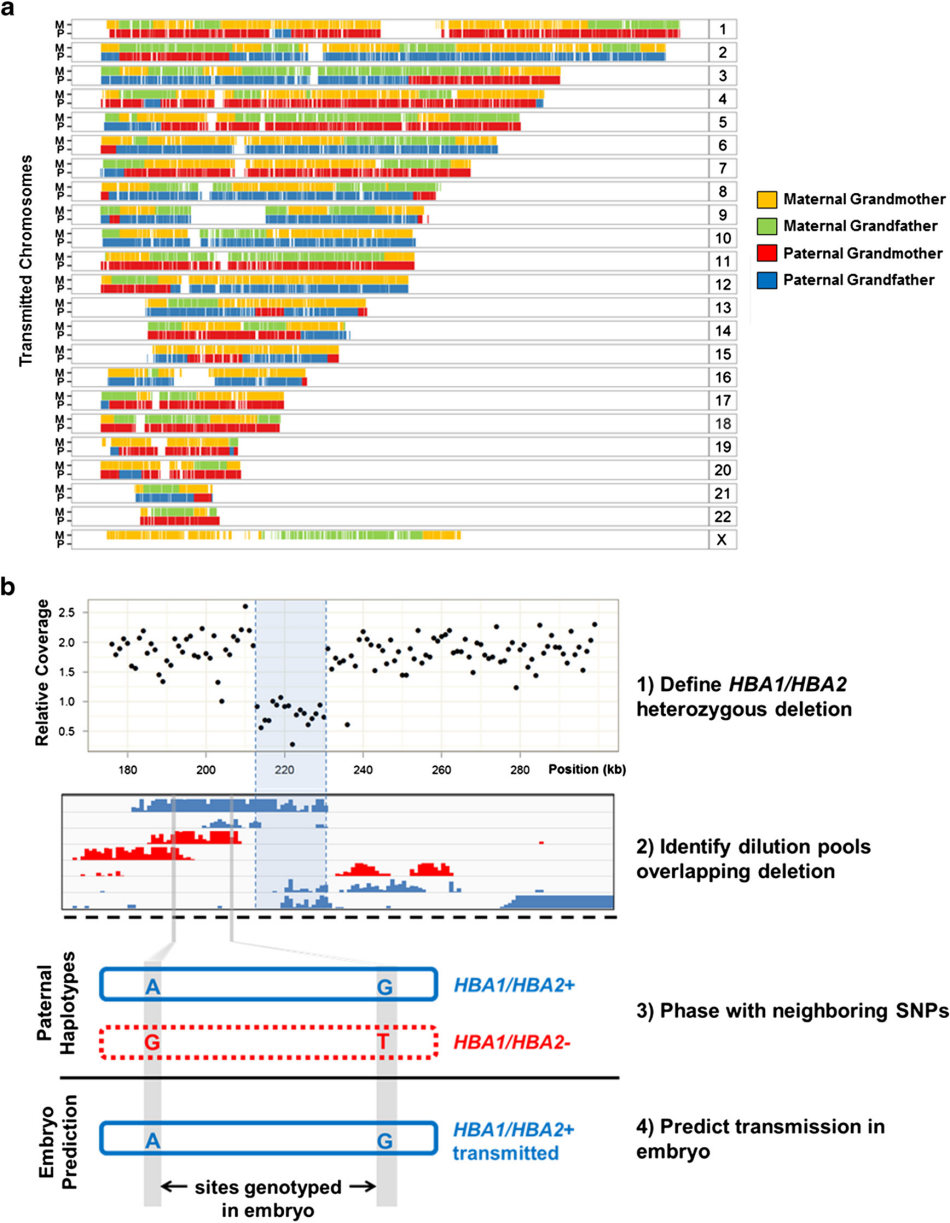
Results of embryo predictions. **(a)** Predicted transmission of parental haplotypes to the embryo after rapid array-based genotyping with ‘Parental Support’. A hidden Markov model incorporating parental phase and SNP array measurements of an embryo was used to obtain the probability of each parental haplotype being transmitted into each embryo. These states and state probabilities were used to determine which parental haplotypes composed the embryo genome and to define the location of meiotic recombination events. The example shown here is colored according to grandparental haplotype after incorporating SNP array measurements from grandparents within predictions. M: Maternal; P: Paternal. **(b)** Phasing of *HBA1/HBA2* deletion in the embryo’s father via dilution pool sequencing. The deletion was identified using whole genome sequencing (WGS). The *HBA1/HBA2* containing haplotype was determined by identifying multiple dilution pools that overlapped the deleted region (in blue). Transmission of this haplotype was predicted by comparing informative PS embryo genotyping sites (blue letters) with corresponding sites within parental haplotypes.

To determine accurate embryo genotypes, we developed an approach we term ‘Parental Support’, which is an informatics technique that uses relatives’ samples including any combination of parents, siblings, and sperm to infer genotypes [24,27]. In this case, we combined SNP array measurements from parents and sibling embryos with recombination frequencies [27] in a statistical model to determine the Maximum Likelihood Estimate (MLE) of phase for heterozygous SNVs in each parent, transmission of parental haplotypes to each embryo, and the location of possible sites of meiotic recombination (Additional file 1: Figures S1 and S2) [24,25,28,29]. This approach results in accurate inference of transmission to the embryo for approximately 120,000 parentally heterozygous sites, hereafter referred to as ‘Parental Support’ (PS) embryo genotypes (Figure 2a and Table 1).

**Table 1:** **Summary of haplotype sources and the number of variants phased/predicted**

**Technique**		**Mother**	**Father**	**Mother (% of all het variants)**	**Father (% of all het variants)**
‘Parental Support’ embryo genotypes	Phased	75,869	73,622	3.9%	3.9%
	Phased and predicted	73,003	71,362	3.8%	3.8%
(+) Phasing by dilution pool sequencing	Phased	1,829,314	1,751,004	94.2%	92.4%
	Phased and predicted	1,672,932	1,589,836	86.2%	83.9%
(+) Phasing by population reference panel	Phased	1,840,200	1,895,730	97.1%	97.6%
	Phased and predicted	1,732,641	1,775,614	91.4%	91.5%
(+) 10× sequence from grandparents	Phased	1,922,421	1,875,363	99.0%	98.9%
	Phased and predicted	1,880,092	1,847,297	96.8%	97.5%

Sites determined by comparison with Illumina trio sequencing (including the offspring) to have poor genotype quality scores or genotypes that violated Mendelian inheritance were discarded for the purpose of evaluating accuracy.

We next sought to incorporate information from haplotype-resolved genome sequencing of both parents. To discover transmissible variants, we first performed shotgun sequencing of the mother and father to 34× and 30× median fold coverage, respectively (Additional file 1: Figure S3a and Table S2). Next, by sequencing haploid subsets of genomic DNA obtained via *in vitro* dilution pool amplification, we directly phased 94.2% of 1.94 million heterozygous SNVs in the mother and 92.4% of 1.89 million heterozygous SNVs in the father into long haplotype blocks [11,14-16,22,30]. To further improve parental haplotypes, we incorporated population-based phase using BEAGLE [31] and sequencing data from the 1000 Genomes Project [32] in a manner similar to local conditional phasing in Selvaraj *et al*. [18]. This approach increased the proportion of SNVs phased to 97.1% in the mother and 97.6% in the father.

We then combined PS embryo genotypes with parental haplotypes to predict inherited variation, genome-wide, in the embryo (Additional file 1: Figure S3b). We inferred transmission of parental haplotype blocks based on the intersection of the sites contained in these blocks and sites called by PS embryo genotypes. Using this approach, transmission of 28,402 blocks including 83.9% of paternal SNVs and 25,733 blocks including 86.2% of maternal SNVs were successfully predicted. The addition of population-based phasing estimates resulted in a 4% to 10% increase in the number of SNVs predicted, such that after this step, transmission of 18,850 blocks representing 91.5% of paternal SNVs and 17,233 blocks representing 91.4% of maternal SNVs were predicted.

A small minority of haplotype blocks appeared to be only partially transmitted (419 blocks; 2.2%). While some of these may correspond to meiotic recombination events within parental gametes, most are likely switch errors in the parental haplotypes that resulted from unresolved collisions within the process of dilution pool sequencing (Additional file 1: Figure S4). We corrected for these cases by splitting each affected block at the site of the switch and inferring transmission of the resulting blocks individually.

We assessed the accuracy of our approach by comparing these predictions against the embryo’s true genotypes, as determined by whole genome sequencing of saliva from the resulting newborn. We excluded sites with poor genotype quality scores in any individual within the trio, as well as sites that violated Mendelian inheritance, leaving a total of 3.19 million sites heterozygous within one or both parents (number of heterozygous sites: 1,297,649 maternal-only, 1,251,550 paternal-only; 643,886 both) (Table 1). A total of 312,698 (9.8%) sites were unable to be called in the embryo because transmission could not be predicted from one or both parents. Of the remaining 2.88 million sites (90.2%), we successfully predicted the embryo genotype with 99.5% accuracy (Tables 1 and 2). If we restrict our predictions to sites where only one parent is heterozygous, 90.9% of paternal-only heterozygous sites and 91.0% of maternal-only heterozygous sites were predicted, with 99.5% accuracy (Additional file 1: Table S3).

**Table 2:** **Prediction accuracy for embryo genotypes**

**Individual**	**Site**	**Other parental genotype**	**Number of sites**	**Accuracy**
Father	Heterozygous, predicted	Homozygous	1,138,851	99.5%
		Heterozygous, predicted	561,745	99.5%
		Heterozygous, unpredicted	32,045	NA
	Heterozygous, unpredicted	All	112,699	NA
Mother	Heterozygous, predicted	Homozygous	1,179,791	99.5%
		Heterozygous, unpredicted	34,078	NA
	Heterozygous, unpredicted	Homozygous	117,858	NA
		Heterozygous, unpredicted	16,018	NA

Transmission predictions for this analysis made use of PS embryo genotypes as well as molecular haplotyping and population-phasing of the parental genomes, but did not make use of grandparental genotypes. Accuracy defined as the percentage of transmitted alleles correctly predicted, out of all predicted sites. Sites determined by comparison with Illumina trio sequencing (including the offspring) to have poor genotype quality scores or genotypes that violated Mendelian inheritance were discarded for the purpose of evaluating accuracy. A total of 312,698 sites were omitted due to unsuccessful phasing in either parent (sum of categories above with ‘NA’ in accuracy column).

NA: not applicable.

Among erroneously predicted sites, 57% (n = 8,421) occurred in ‘clusters’ of two or more adjacent sites, suggesting that most inaccuracies result from improper parental phasing or incorrect prediction of meiotic breakpoints. Sites for which we were unable to make predictions failed at different stages of analysis (Additional file 1: Figure S5) with the majority (72%, n = 227,675) phased into haplotype blocks but not predicted with respect to transmission due to sparse measurement in the embryo. Prediction of these sites could be improved either by increasing the size of parental haplotype blocks or by increasing the number of measurements made on the embryo (for example, using denser SNP arrays). To estimate the potential for improvement by using a higher density SNP array, we simulated the discovery of additional embryo genotypes and repeated our predictions. We estimated that increasing the number of PS embryo genotypes consistent with the use of the Illumina 1 M array would boost coverage from 90.2% to approximately 94% (Additional file 1: Figure S6).

To investigate how predictions would improve with the availability of grandparental genotypes, we sequenced the genomes of the embryo’s four grandparents to 10× depth. We phased each parent’s genome using variants called from shotgun sequencing of maternal and paternal grandparents and combined the resulting haplotypes with the blocks determined by *in vitro* dilution pool phasing. The inclusion of grandparental genotypes increased the number of positions for which predictions could be made in the embryo to 3.10 million sites (97.2%) with a slight decrease in accuracy from 99.5% to 99.3% (possibly due to errors introduced when phasing using low-coverage grandparental genomes). If we restrict our predictions to sites where only one parent is heterozygous, 97.6% of paternal-only heterozygous sites could be predicted with 99.4% accuracy, while 96.8% of maternal-only heterozygous sites could be predicted with 99.3% accuracy (Additional file 1: Table S3).

Both parents were carriers of a 40-kb deletion at the alpha hemoglobin locus (*HBA1/HBA2*), and underwent PGD testing to select for embryos carrying only non-deletion alleles. The clinical test was originally performed by phasing the deletion to a haplotype using DNA from grandparents and subsequently inferring which parental haplotype was transmitted to the child. In our approach, we set out to predict transmission in the absence of grandparental haplotypes, both at the *HBA* locus and genome-wide. We first phased the deletion in each parent with neighboring SNVs [23]. We manually selected haplotype blocks overlapping the deletion and identified neighboring heterozygous SNVs in these blocks that were also genotyped within the embryo (Figure 2b). Both parents transmitted SNVs that were linked to intact *HBA1* and *HBA2* haplotypes, thus we predicted that the embryo did not inherit the *HBA1/HBA2* deletion from either parent. Our results were consistent with the clinical PGD prediction and with the genome sequence of the resulting newborn (Additional file 1: Table S4).

## Discussion

We present an approach to predict the inherited whole genome sequence of a human embryo through a combination of rapid genotyping of multiple embryo biopsies and haplotype-resolved parental genome sequencing. The types and quantities of materials used are consistent with those routinely collected in a clinical PGD setting. Importantly, all embryo genotyping data used in this study were obtained using a rapid genotyping protocol that is already in use for chromosomal screening (Additional file 1: Figure S7) and haplotype-resolved parental genome sequences can readily be determined in advance of PGD [6].

From a technical perspective, the prediction of an embryo genome as described here differs in several key ways from the prediction of a fetal genome through sequencing of maternal plasma [33,34]. Specifically, fetal genome prediction involved millions of noisy but potentially informative measurements that are the result of sequencing a mixture of fetal and maternal cell-free DNA fragments. In contrast, the approach described here uses products of whole genome amplification that have been genotyped using SNP arrays and thus results in fewer informative measurements - about 50,000 to 75,000 informative SNVs within each embryo. Additionally, single gene PGD involves biopsy of multiple embryos per cycle (average n = 5.9 according to a recent study) and our approach uses these sibling embryos to phase parental genomes [35]. Fresh embryo transfer requires a rapid (24-h) turnaround for any genomic assay although a trend towards frozen embryo transfer could alleviate that restriction. Finally, and perhaps most importantly, the sparseness of embryo biopsy genotype estimates is such that it is critical that parental haplotype phase estimates are highly accurate (that is, with few switch errors).

The ability to infer inherited variation genome-wide with high accuracy and completeness could have profound implications for the future of PGD. A growing area in PGD relies on testing for the presence of single gene Mendelian disorders within embryos. While flexible methods such as karyomapping are gaining in use, these require related samples such as grandparents or other family members/specimens to phase variants at the outset [19,36,37]. The inferred genome sequence of an embryo could potentially be used to simultaneously test for all inherited Mendelian diseases for which the genetic basis is well understood, as well as digenic and multigenic disorders like Bardet-Biedl syndrome and Hirschsprung’s disease. Additionally, although many challenges remain, we are increasingly able to predict multifactorial disease risk from genetic information [38-40]. However, as large numbers of variants contribute to complex disease, genome-wide analysis is likely to be critical for risk prediction. We note that because haplotypes can be determined experimentally, our approach does not require grandparental DNA, enabling broader application of this technology, although the comprehensiveness of predictions clearly increased when it is available.

There remain several major limitations and key avenues for improvement. First, somatic mosaicism within an embryo (primarily with respect to karyotype) can confound genome predictions, and thus the genome of an embryo biopsy may not fully represent the genome of the resulting fetus. Second, our approach does not detect *de novo* copy number alterations or *de novo* point mutations that arise within the parental gametes. Large *de novo* copy number alterations can potentially be identified from SNP array data, although this was not attempted in this study. While this work was in review, Peters *et al.* published a related method that uses dilution pool sequencing of cells from trophectoderm biopsies to determine more than 80% of *de novo* mutations in an embryo with high accuracy [41]. The approach is very compelling, but the time and labor required after embryo biopsy necessitates embryo freezing. By contrast, our approach can work with both fresh and frozen embryos and does not necessarily require any changes to a clinically available PGD procedure.

Another limitation is that although we were able to predict approximately 3 million sites in the embryo with high accuracy, we were unable to make predictions at over 300,000 sites. These sites were missed primarily due to incomplete parental haplotypes but also the relative sparseness of embryo genotyping. As technologies for haplotype-resolved genome sequencing continue to improve, for example, [17], and as denser embryo genotyping is performed, we anticipate that a higher proportion of the embryo genome will be predictable by this general strategy. The cost of this method remains prohibitive for routine clinical use, especially when compared with methods that rely on more conventional molecular techniques (for example, PCR, FISH, arrayCGH), largely due to the cost of whole genome sequencing and haplotyping each parent (Additional file 1: Table S5). Finally, multiple clinical studies with many more samples are needed to fully validate this method for whole genome prediction.

Nonetheless, this proof-of-concept was completed using tools and methods that are readily available. Genotyping arrays are in routine use for chromosome screening of embryos following IVF. Similarly, technologies for performing haplotype-resolved genome sequencing are proliferating and maturing, with steady improvements in the length and accuracy of the resulting haplotype blocks. Modest improvements in either protocol will likely increase the accuracy and coverage of our predictions, and the costs of whole genome sequencing are more generally expected to continue to fall.

## Conclusions

In this study, we introduce a method for predicting the genome of an embryo by combining haplotype-resolved genome sequencing of parents with rapid embryo genotyping. We use this approach to predict the transmission of approximately 3 million paternally and/or maternally heterozygous sites with greater than 99% accuracy. We also predicted the transmission of a 40 kb *HBA1/HBA2* deletion in the embryo. This approach extends preimplantation screening to potentially include all Mendelian disorders as well as complex diseases with a defined genetic basis. Importantly, our approach does not require substantial changes to the current practice of preimplantation genetic diagnosis. As sequencing methods continue to improve, we anticipate that this approach could be a valuable addition to preimplantation testing.

## Additional file

Additional file 1: **Supplementary materials.** This file describes a detailed description of the bioinformatics methods and experimental work used in this study.
