# Supplementary material for: Whole genome prediction for preimplantation genetic diagnosis
Source: Genome Med. 2015 Apr 8;7(1):35. doi: 10.1186/s13073-015-0160-4 (PMC4445980; doi:10.1186/s13073-015-0160-4)
Supplement: Supplementary file 1 — Supplementary materials. This file describes a detailed description of the bioinformatics methods and experimental work used in this study. [file 13073_2015_160_MOESM1_ESM.docx]

**Additional file 1**

**Supplementary Materials for “Whole Genome Prediction for Preimplantation Genetic Diagnosis”**

[Supplemental Notes 2](#_Toc409547915)

[Note S1: Parental haplotype resolution via *in vitro* dilution sequencing. 2](#_Toc409547916)

[Note S2: Haplotype construction from dilution pool sequences. 2](#_Toc409547917)

[Note S3: Local statistical phasing using 1000 Genomes data. 3](#_Toc409547918)

[Note S4: Maximum likelihood estimate of parental haplotypes (Parental Support). 4](#_Toc409547919)

[Note S5: Hidden Markov Model to predict transmission of parental haplotype to embryo (Parental Support). 5](#_Toc409547920)

[Note S6: Incorporation of grandparental genotypes. 8](#_Toc409547921)

[Supplemental Figures 9](#_Toc409547922)

[Figure S1: Overview of Parental Support (PS) approach. 9](#_Toc409547923)

[Figure S2: HMM probability of each paternal haplotype being transmitted across chromosome 1 for use in determining PS embryo genotypes. 10](#_Toc409547924)

[Figure S3: Combining parental haplotype blocks with PS embryo genotypes to infer embryo genome. 11](#_Toc409547925)

[Figure S4: Explanation of dilution pool phasing and description of switch error. 12](#_Toc409547926)

[Figure S5: Sources of dropout in predictions. 13](#_Toc409547927)

[Figure S6: Effect of increasing SNP array density on prediction coverage. 14](#_Toc409547928)

[Figure S7: A potential clinical workflow. 15](#_Toc409547929)

[Supplemental Tables 16](#_Toc409547930)

[Table S1: Summary of sequencing/array measurements.. 16](#_Toc409547931)

[Table S2: Sequencing statistics of genomes. 17](#_Toc409547932)

[Table S3: Accuracy of embryo genome predictions. 18](#_Toc409547933)

[Table S4: Prediction of aneuploidy across embryos and comparison with Parental Support predictions. 19](#_Toc409547934)

[Table S5: Estimate of costs involved in this study. 20](#_Toc409547936)

Supplemental Notes

Note S1: Parental haplotype resolution via *in vitro* dilution sequencing.

We used a modified “Long Fragment Read” protocol to perform haplotype-resolved genome sequencing of each parent [[11](#_ENREF_11)]. Briefly, high molecular weight DNA was diluted to an average concentration of 4 pg/μL and placed within a 96-well plate. Each diluted pool was subjected to amplification using phi29 (Enzymatics) with a low concentration of dUTP (10 μM). Sequencing libraries were prepared as previously described in Neiman *et al.* [[30](#_ENREF_30)] with some modifications. Amplified DNA fragments were using USER enzyme. 2 μL of the product of phi29-amplification was added to 0.1 μL of USER enzyme (0.1 U/μL), 1 μL of 10x T4 DNA ligase buffer (NEB) and molecular biology-grade water for a total of 10 μL. For digestion, this mixture was incubated for 1 hour at 37°C and 15 minutes at 65°C. For the end-polishing, phosphorylation and adenylation phase, we added 5 nanomoles each dNTPs (NEB), 3 μL 10x ligase buffer (NEB), 1 μL 10% PEG-4000, 0.1 μL of DNA Polymerase 1 (10U/μL), 0.5 μL of T4 PNK (10U/μL, NEB), 0.2 μL Taq DNA Polymerase (5U/μL, NEB) and molecular biology grade water to 30 μL for a total reaction volume of 40 μL. This reaction was incubated for 30 min at 37°C and 20 minutes at 72°C with a final incubation at 4°C. Following this step, to each reaction we added 1μL 10x T4 Ligase Buffer w/ATP (NEB), 1 μL of 10μM Library Adaptors and 1 μL of NEB T4 DNA Ligase (400U/μL, NEB) and molecular biology grade water to 10 μL for a total reaction volume of 50 μL. The ligation step was carried out at 16°C overnight in a pre-cooled thermal cycler. After ligation, the samples were bead-purified (AMPure, Agencourt) and prepared for PCR using KAPA HiFi kit. 10 μL of post-ligation product was added to 12.5 μL of 2x Kapa HiFi mastermix (KAPA) as well as 0.3 uM of each primer under the following conditions: 95°C for 3 min, 10 cycles of 98°C for 20 seconds, 65°C for 15 seconds, 72°C for 40 seconds followed by 72°C for 2 minutes. Following PCR amplification libraries were pooled, bead-purified (AMPure, Agencourt) using a 1:1 ratio of beads to sample, and sequenced.

Note S2: Haplotype construction from dilution pool sequences.

Haplotype blocks were constructed from dilution pool sequences using a two-step approach. First, initial fragment boundaries were defined using the samtools targetcut function with settings -i 6000. Basecalls were made within fragments at sites determined to be heterozygous by whole genome sequencing. Second, these initial fragments were split at those sites observed to be heterozygous from dilution pool sequences (allele frequency between 0.25 and 0.75). Resulting haplotype fragments were assembled into blocks using the RefHap haplotype assembly package with default settings.

Note S3: Local statistical phasing using 1000 Genomes data.

To improve block contiguity and phase predictions, we performed additional phasing using haplotypes from 1000 Genomes [[32](#_ENREF_32)]. Phased genotype calls from 379 individuals of European descent from version 3 of the 1000 Genomes integrated variant call set [[32](#_ENREF_32)] were merged with unphased genotype calls from the mother and father in the present study. This union set of variant calls was filtered to remove indels, sites missing in 1000 Genomes, and 1000 Genomes singleton and doubleton sites. The resulting set of variants was phased by BEAGLE version 4 r1182 [[31](#_ENREF_31)] with options “phase-its=40 usephase=true dump=[file]”. To integrate population-level phasing with haplotypes obtained by dilution pool sequencing, we created “blocks” of haplotypes determined by population-based phasing. The boundaries and phase of haplotype blocks were determined using the intermediate haplotypes generated by BEAGLE (after burn in) in the following manner:

For a given pair of variants at positions (A and B) in the maternal or paternal genome, a pairwise confidence score was estimated by determining the proportion of intermediate sampled haplotypes in which phase between minor alleles at these sites was concordant. At each position A and B we observe two alleles (A_1_ and A_2_ for position A as well as B_1_ and B_2_ at position B) across each of the iterations from BEAGLE. We defined a confidence metric C as:

$$C=\frac{{(x_{11}-p_{1}q_{1})}^{2}}{(p_{1}p_{2}q_{1}q_{2})}$$

Where p_1_ is the frequency of A_1_, p_2_ is the frequency of A_2_, q_1_ is the frequency of B_1_, q_2_ is the frequency of B_2_, and x_11_ is the frequency of seeing A1 and B1 within a given iteration.

We calculate this metric across for each SNV and each of the 3 SNVs that are immediately subsequent and adjacent to the first. Positions for which C is less than a threshold value (0.97) are used to define boundaries of haplotype blocks. These blocks were also required to contain at least 3 phased SNVs. Within each block, the phase of variants was set as the relative phase determined by the final iteration of BEAGLE’s haplotype. These haplotype blocks were subsequently assembled with blocks obtained by dilution pool sequencing using the RefHap haplotype assembly package.

Note S4: Maximum likelihood estimate of parental haplotypes (Parental Support).

As part of the “Parental Support” process to obtain accurate embryo genotypes, parent haplotypes were estimated from embryo measurements by combining SNP array measurements from multiple embryos and from the parents along with recombination frequencies from the HapMap database [[25](#_ENREF_25)]. We describe the process in full within US Patent 8515679_B2 [[24](#_ENREF_24)], and a brief summary follows. Array measurements from euploid embryos and parents were entered into our model as separate inputs. We estimate likely genotypes (AA, AB/BA, BB) of each parent as a function of measurements of A and B channel intensities on the array. Embryo genotypes are estimated similarly with an additional parameter of “allele dropout” defined as the probability of one present copy of an allele failing to produce a measurement above the noise floor (calculation shown below). The algorithm works by developing a joint probability distribution for the assignment of genotypes to all of the samples, parents and embryos, simultaneously (**Additional file 1: Figure S1**). This statistical model incorporates the fact that an embryo will inherit alleles from the same parent homolog on consecutive SNPs, unless a meiotic recombination (with probability estimated in the HapMap database) has occurred between the two SNPs. The joint distribution on genotype probabilities thus combines the array data, the individual sample genotypes suggested by the array data, and the parent haplotyping that could produce those distributions of genotypes among various embryos. Summing over the possible embryo genotypes allows probabilities to be assigned to phased parent genotypes AB and BA, where the alleles are ordered according to their position on haplotype 1 or 2. These phased SNPs are used to construct a haplotype. Our algorithm operates on a region of approximately 1000 consecutive SNPs, with full-chromosome haplotypes constructed by assembling regions using 200 SNP overlaps.

Note S5: Hidden Markov Model to predict transmission of parental haplotype to embryo (Parental Support).

SNP array data from an individual embryo was analyzed to estimate the positions of meiotic recombination events, thereby determining throughout the genome which haplotype was inherited from each parent. In addition to embryo SNP array intensities, parent phasing results are an input into this calculation. First, data from all chromosomes is combined to estimate an initial estimate of the allele dropout rate using the Baum-Welch algorithm [[29](#_ENREF_29)]. Note that allele dropout rates close to 50% are commonly observed in array data with low DNA input amount, as is the case of embryo array data. This implies that a heterozygous SNP will appear homozygous approximately half the time and explains why direct genotyping of single-cell data is not effective. The estimated parameters are then used to create a hidden Markov model for the transmission of each parental haplotype, with a separate model being created for each parent. The following paragraphs provide further details of the modeling approach (**Additional file 1: Figure S1**). To simplify our discussion we restrict our discussion to inheritance of maternal haplotypes, with inheritance of paternal haplotypes calculated in a similar way.

A hidden Markov model structure is defined in order to estimate the parent haplotype state at each SNP. We begin by defining the concept of parental genotype context (table below), assuming that phased parent genotypes of the form AB and BA are available.

| **Context** | **Mother** | **Father** | **Allele queried** | **Purpose** |
| --- | --- | --- | --- | --- |
| **No Signal** | AA | AA | B | Calculate noise floor |
| **No Signal** | BB | BB | A |  |
| **Polar** | AA | BB | A | Calculate allele dropout |
| **Polar** | BB | AA | B |  |
| **I_1_** | AB | BB | A | Used in HMM |
| **I_1_** | BA | AA | B |  |
| **I_2_** | BA | BB | A |  |
| **I_2_** | AB | AA | B |  |

First define the no-signal context as the set of measurements where there is no signal expected in the embryo measurement. Specifically, these are measurements on the B channel for SNPs where the parent genotypes are both AA, and measurements on the A channel for SNPs where the parent genotypes are both BB. The first model parameter calculated for an embryo is the noise floor of its array measurements, which is defined as the 95^th^ percentile SNP measurement from the no-signal context. We consider an allele to be detected in the array data if the corresponding intensity measurement is greater than the noise floor. As a consequence of the definition, there is a 5 percent probability that an allele which is not actually present will be detected.

Next define the polar context as the set of measurements where signal is expected from exactly one copy of the measured allele regardless of parent haplotype state. These are the set of measurements where one parent has genotype AA and the other parent has genotype BB, measured on either channel. The allele dropout rate (d) is defined as the probability of one present copy of an allele failing to produce a measurement above the noise floor, or “dropping out” in the measurement. The allele dropout rate can be estimated (in a simplified method) as the fraction of polar context measurements with intensity less than the noise floor. Note that when two copies of an allele are present they are considered to drop out independently, and so the probability of a dropout is d^2^.

In order to estimate the model parameters, the array intensities are first converted to a binary indicator of allele presence or absence for each allele at each SNP. The hidden Markov model for each parent estimates probabilities on the set of parent haplotype states. The main parent haplotype states are h0, h1, and h2. Lack of any haplotype from the parent is indicated by the h0 state, which would indicate a deletion. Presence of haplotype one is indicated by the h1 state, and similar for haplotype two. Aneuploidy states were considered as well [[24](#_ENREF_24)], but are not described here. The table below shows the probability of failing to observe an allele (P_d_) in a particular parent genotype context, conditioned on a particular parent haplotype state. We introduce two new parent contexts: I_1_ and I_2_. Indicator context I_1_ is defined as the set of measurements where exactly one copy of the allele is present if the state is h1 and no copy of the allele is present otherwise. For example, the A allele measurement for the case of (mother: AB, father: BB) and the B allele measurement for the case of (mother BA, father: AA) are part of the I_1_ indicator context for the mother.

|  | | **Emission probability do not observe allele (P_d_)** | | **Emission probability observe allele (1-P_d_)** | |
| --- | --- | --- | --- | --- | --- |
| **State** | **Description** | **I_1_** | **I_2_** | **I_1_** | **I_2_** |
| h0 | no haplotype present from parent | 0.95 | 0.95 | 0.05 | 0.05 |
| h1 | only haplotype 1 present | d | 0.95 | 1-d | 0.05 |
| h2 | only haplotype 2 present | 0.95 | d | 0.05 | 1-d |

The emission probability of an observation conditioned on the hidden state of the parent haplotype is thus either the context-dependent P_d_ or its complement (1-P_d_), depending on whether or not the allele is detected. The transition probability of switching from one state to another was set as a constant to approximate the recombination probability between SNPs. Given the structure of the transmission model and the state transition model, the maximum-likelihood values of model parameters are estimated using the Baum-Welch algorithm[[29](#_ENREF_29)]. Two sets of parameters are estimated for each embryo: one set for the haplotype inheritance from each parent. The table below shows the estimated model parameters for the embryo of interest.

| **Parameter** | **Additional information** | **Value** |
| --- | --- | --- |
| allele dropout rate | maternal haplotype | 0.198 |
| allele dropout rate | paternal haplotype | 0.196 |
| transition probability | maternal haplotype | 0.0039 |
| transition probability | paternal haplotype | 0.0023 |
| noise floor | A channel measurement | 1669 |
| noise floor | B channel measurement | 3074 |

The haplotype state estimates for each chromosome can then be estimated from the hidden Markov model. The forward-backward algorithm [[29](#_ENREF_29)] calculates the probability of each haplotype state at each SNP, conditioned on all of the observations over the chromosome. A recombination in a euploid embryo is indicated by a switch from haplotype one to haplotype two, or the reverse. One could consider a confidence threshold such as 0.99 and conclude that the recombination happened between the SNP where the confidence on the first state fell below 0.99 and the SNP where the confidence on the second state rose above 0.99 (**Additional file 1: Figure S2)**.

We determined “Parental Support” (PS) embryo genotypes by combining MLE estimates of parental phase and the HMM-predicted meiotic breakpoints discussed above. We used a likelihood threshold of 0.95 to determine which parental positions to consider, and a HMM-estimated probability of 0.99 to define the meiotic breakpoints in the embryo (and thus the resulting genotypes of the embryo).

Note S6: Incorporation of grandparental genotypes.

To examine the effect of grandparental genotypes on our predictions of embryo genome maternal and parental grandparents were sequenced to ~10x depth. Parental and grandparental heterozygous variant calls were phased by trio phasing using GATK PhaseByTransmission with default options and resulting variants were filtered for variants with a threshold Phred-scaled phasing quality score > 25. We split resulting haplotypes into fragments containing ten variants each and assembled them with haplotype blocks using RefHap. As trio phasing results in phase across the chromosome, we also incorporated grandparental-phased variants into our predictions of PS embryo genotypes to assist with determining haplotype block transmission.

Supplemental Figures


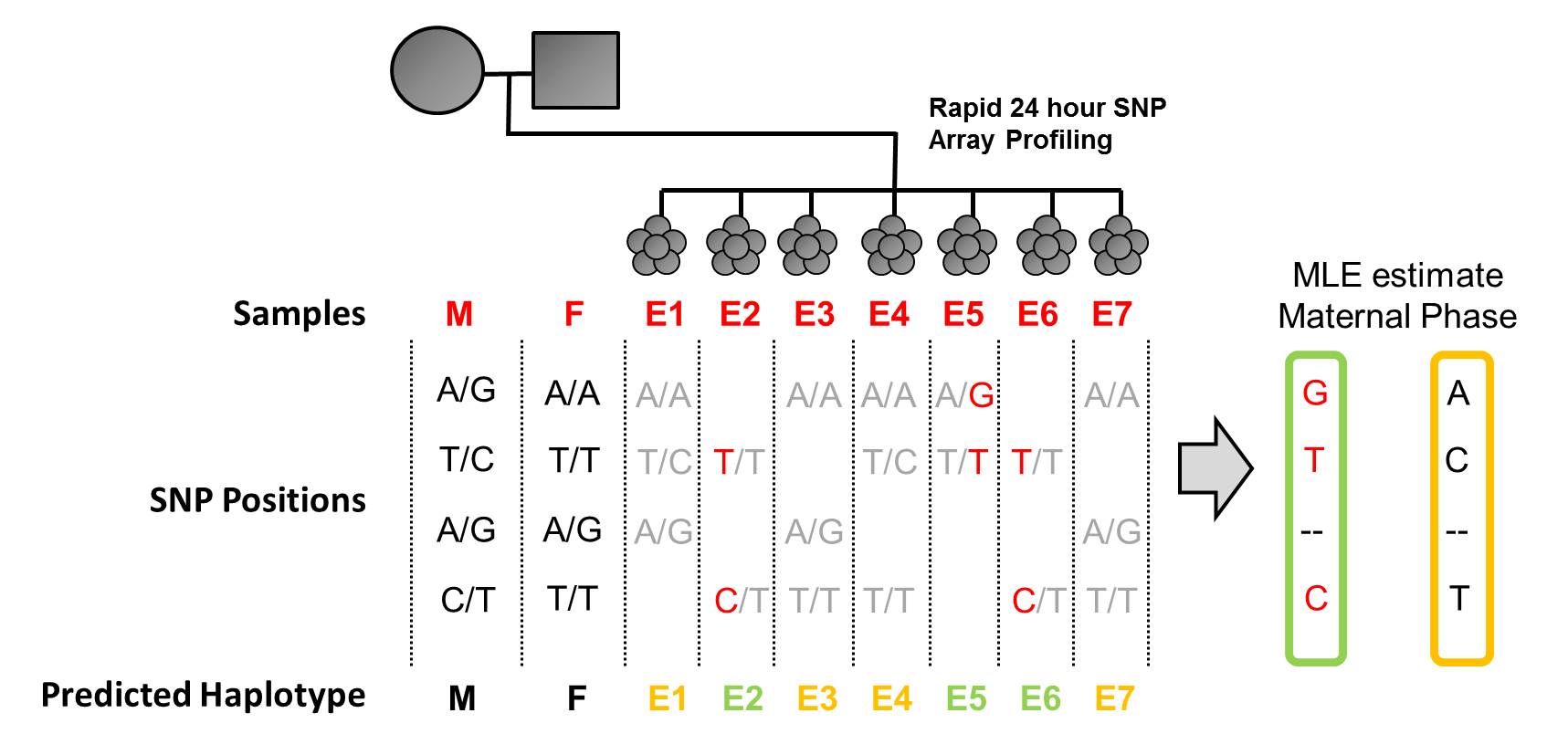


Figure S1: Overview of Parental Support (PS) approach.

Parental genotypes are used in combination with embryo SNP array measurements to simultaneously phase a subset of sites in each parent (MLE estimate) and infer haplotype transmission to embryos (Predicted Haplotype). The resulting embryo genotypes are referred to as “Parental Support” (PS) embryo genotypes in this manuscript. In the simplified example shown, only the mother’s haplotype is considered. Embryo measurements (in gray) are noisy and susceptible to dropout. An allelic dropout rate is measured for each embryo by comparison to parental data (not shown) and used during prediction of parental haplotypes. When inferring haplotype transmission, recombination frequencies from HapMap are used to predict the likelihood of meiotic recombination events (not shown).


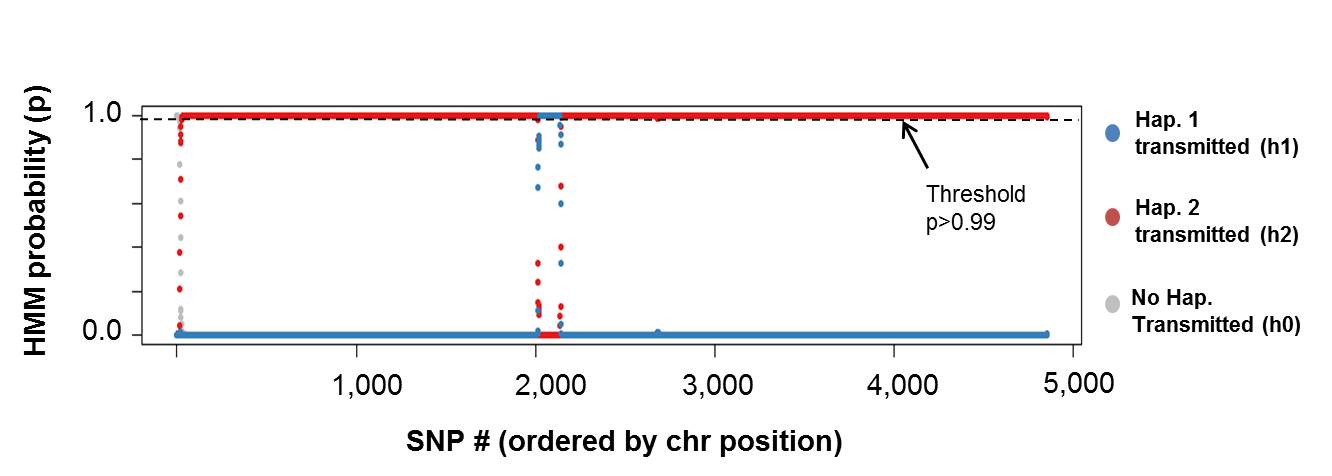


Figure S2: HMM probability of each paternal haplotype being transmitted across chromosome 1 for use in determining PS embryo genotypes.

To determine PS embryo genotypes, we integrated MLE estimates of parental phase with an HMM estimate of which parental haplotype is transmitted. Resulting HMM probabilities of paternal haplotype transmission are shown across 5,000 SNPs of chromosome 1. Each homolog has three possible states (h1, h2 or no haplotypes transmitted). We used a threshold probability of 0.99 (dotted line) to define meiotic breakpoints within an embryo, which makes up the results shown in **Fig. 2a**.


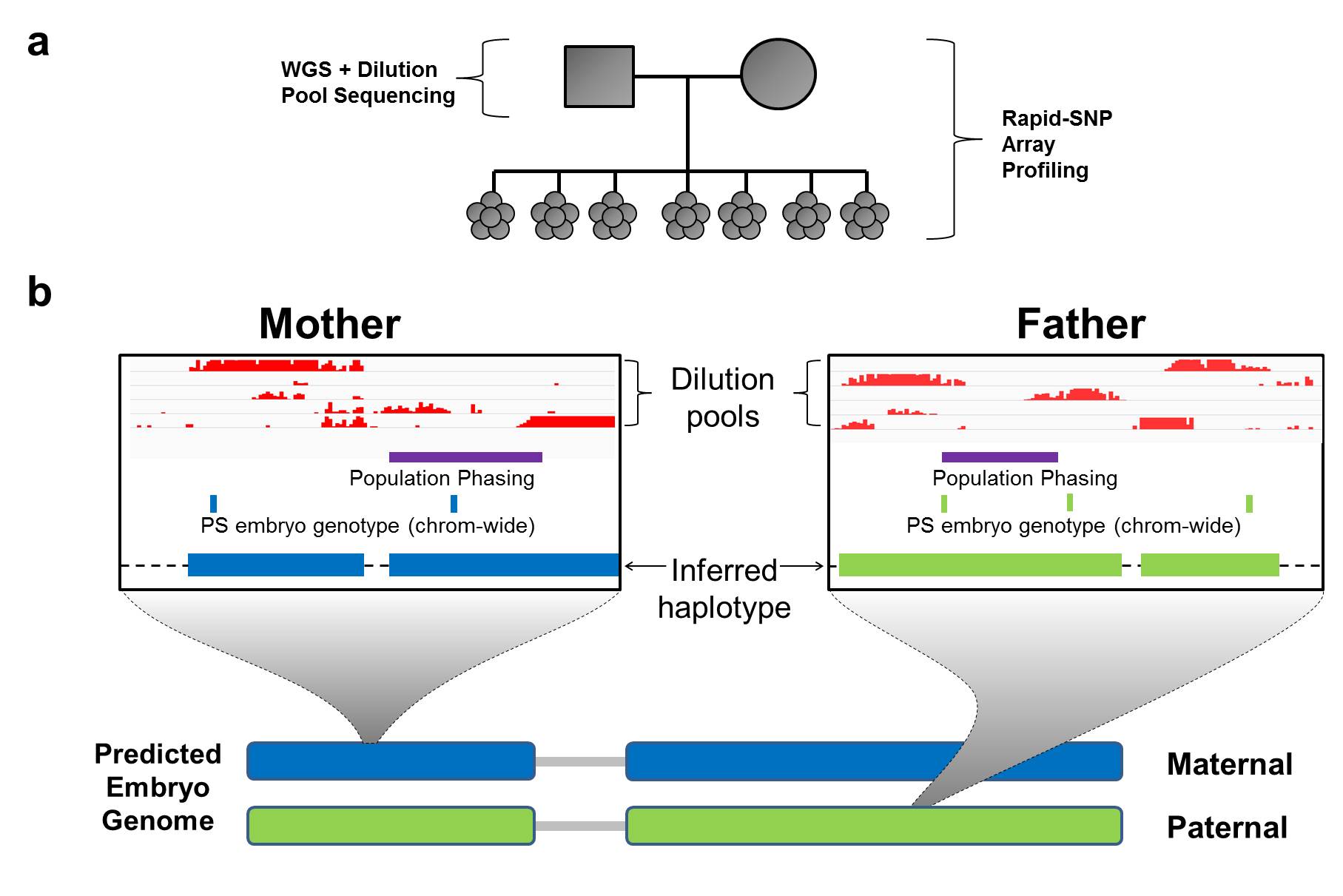


Figure S3: Combining parental haplotype blocks with PS embryo genotypes to infer embryo genome.

**a)** Parents were sequenced using standard shotgun whole genome sequencing and resulting variants were haplotype resolved using a combination of dilution pool sequencing and population-based phasing. All samples (including embryo biopsies) were assayed using a rapid SNP genotyping technology. **b)** To infer the transmission of variants in the embryo, we used parental haplotypes blocks that had been constructed using a combination of dilution pool phasing (in red) and population-based phasing (in purple). Data obtained by SNP array genotyping of embryos and parents (PS embryo genotypes) was used to predict transmission of specific parental haplotype blocks to the embryo.


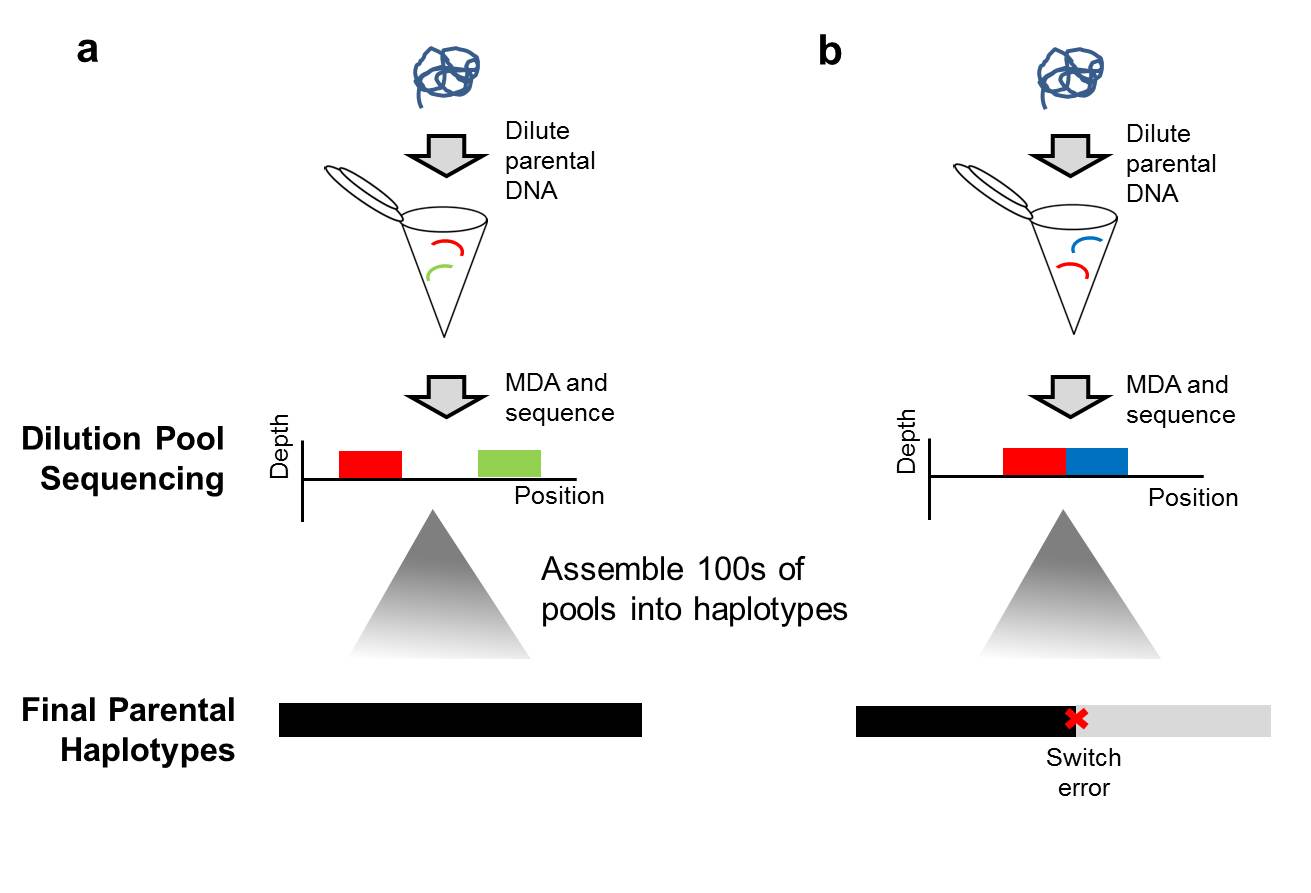


Figure S4: Explanation of dilution pool phasing and description of switch error.

**a)** Dilution pool sequencing operates by diluting parental DNA such that each pool has, on average, a sub-haploid equivalent of DNA. After amplification and sequencing of each pool, the resulting sequence forms “islands” of coverage across the genome corresponding to long (>10 kb) fragments of parental DNA. Sequence from these regions can be assembled into haplotype blocks. **b)** Switch errors can occur when DNA from two overlapping regions is amplified within an individual dilution pool. If the two pieces of DNA are from opposing haplotypes, this DNA results in a switch error within haplotype assemblies. MDA: multiple displacement amplification.


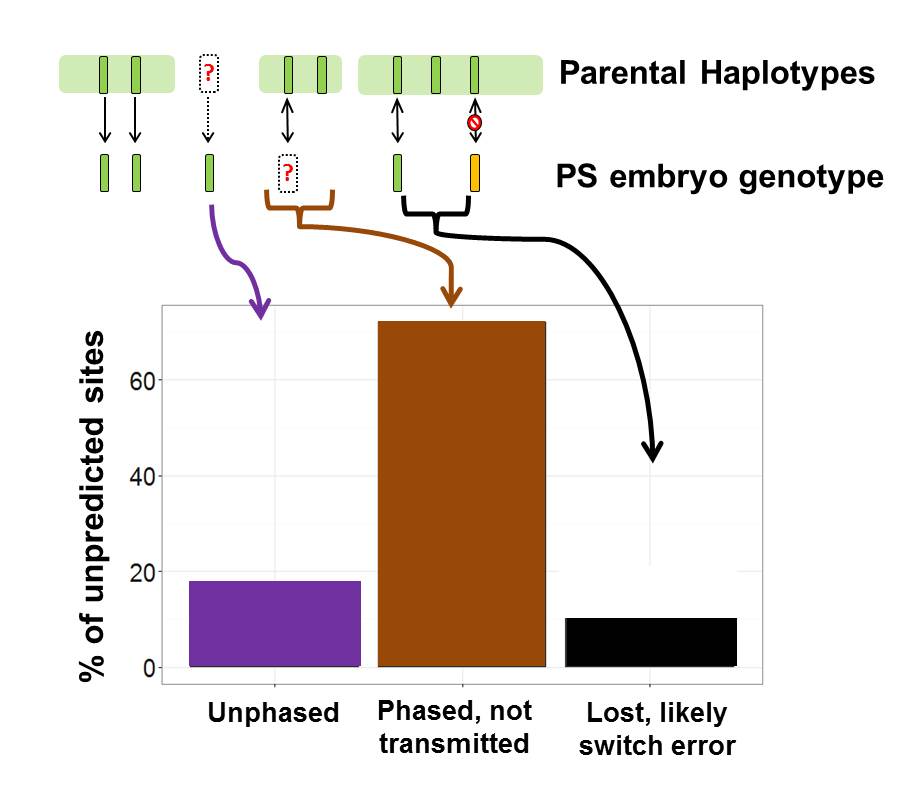


Figure S5: Sources of dropout in predictions.

We were unable to make predictions for a total of 312,698 sites across the embryo genome. 18% of these sites were unable to be phased in the parent(s) using either dilution pool or population-based phasing. 72% of these sites were not able to be predicted due to a lack of overlap of sites within parental haplotype blocks and sites called by “Parental Support” (PS) embryo genotypes. Finally, 10% of these sites were lost in the process of correcting switch errors within parental haplotype blocks.


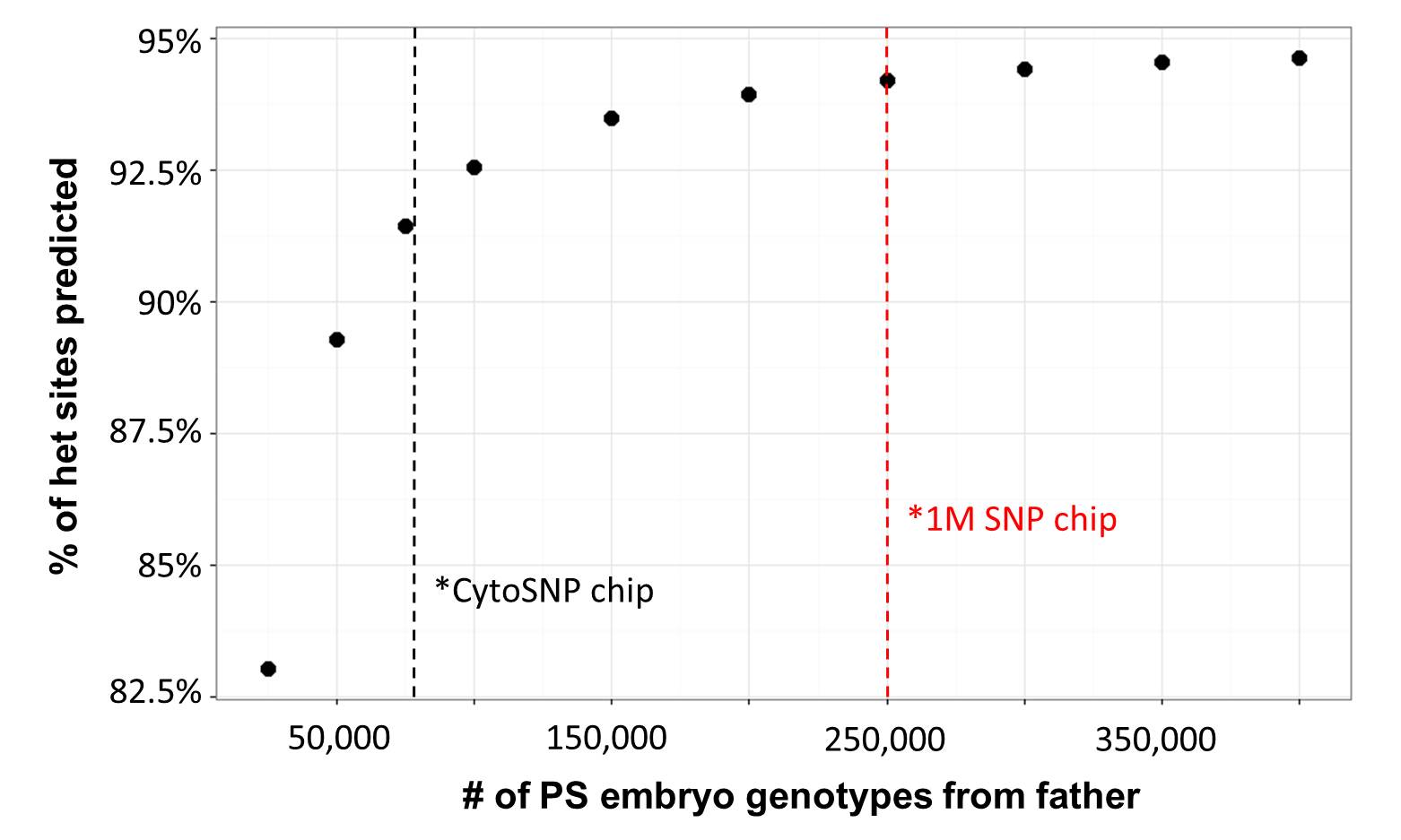


Figure S6: Effect of increasing SNP array density on prediction coverage.

We simulated higher density genotyping of embryos using phased genotypes from whole genome sequencing of the family trio. As expected, the proportion of heterozygous sites at which paternal transmission could be predicted (y-axis) increases with the density of embryo genotype measurements (x-axis) used to predict transmission of paternal haplotypes. A similar finding was seen for maternal transmission (not shown). We simulated the potential improvements from increased genotyping density (e.g., from an Illumina 1M chip) by increasing the number of embryo measurements by ~3.3 fold compared with our data (which used the CytoSNP-12 chip with 300K SNPs). Using the denser 1M chip (with equivalent allelic dropout rates) would increase the prediction rate from 91% to 94%.


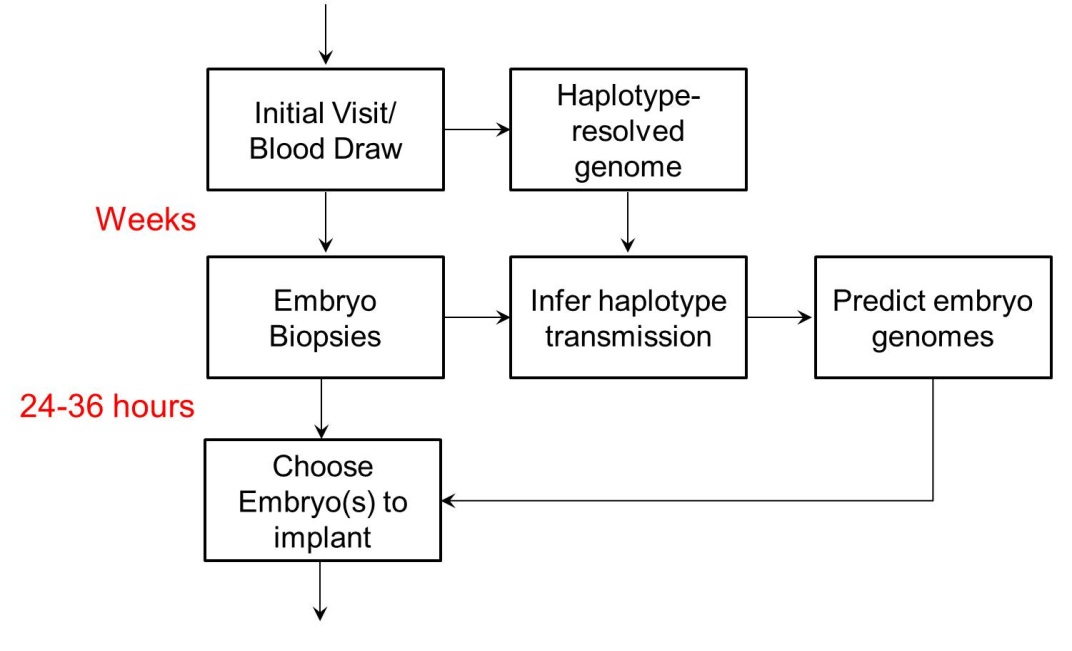


Figure S7: A potential clinical workflow.

Parental haplotyping, via dilution pool sequencing or an alternative method, could be performed after an initial consult at a PGD clinic weeks before an IVF procedure. Rapid genotyping and analysis can take place immediately after embryo biopsy to support fresh embryo transfer.

Supplemental Tables

| **Individual** | **Sample** | **Type of sequencing** | **Purpose** | **Platform** |
| --- | --- | --- | --- | --- |
| Mother and Father | Blood | WGS | Identify variants | Illumina HiSeq |
|  |  | Dilution pool | Phase variants into haplotype blocks | 288 pools MDA followed by HiSeq |
|  |  | Array | Assist in embryo phasing | Illumina CytoSNP |
| Embryo | 1-10 cells | Array | 1. Infer parent phase from multiple embryos 2. Estimate haplotype transmission in single embryo | Illumina CytoSNP |
| Newborn | Saliva | WGS | Validation | Illumina HiSeq |
| Grandparents | Saliva | WGS | Additional phasing | Illumina HiSeq |
|  |  | Array | Assist in embryo phasing | Illumina CytoSNP |

Table S1: Summary of sequencing/array measurements..

WGS: whole genome sequencing; MDA: Multiple Displacement Amplification.

| **Individual** | **Total read pairs** | **Mapping read pairs** | **Final median fold-coverage** |
| --- | --- | --- | --- |
| Mother | 591,877,684 | 551,507,249 | 34 |
| Father | 531,221,020 | 486,817,439 | 30 |
| Newborn | 464,331,229 | 424,553,842 | 28 |
| Maternal GM | 256,207,388 | 157,965,872 | 10 |
| Maternal GF | 463,639,461 | 234,693,301 | 16 |
| Paternal GM | 227,113,806 | 186,704,064 | 12 |
| Paternal GF | 225,078,207 | 176,966,743 | 12 |

Table S2: Sequencing statistics of genomes.

Number of reads and mean coverage across all genome samples sequenced. GM: grandmother. GF: grandfather.

| **Condition** | **Individual** | **Number of sites (het in one parent )** | **% of Sites Predicted** | **Accuracy** |
| --- | --- | --- | --- | --- |
| No Grandparents | Mother | 1,138,851 | 91.0% | 99.5% |
|  | Father | 1,179,791 | 90.9% | 99.5% |
| With Grandparents | Mother | 1,181,754 | 96.8% | 99.3% |
|  | Father | 1,145,496 | 97.6% | 99.4% |

Table S3: Accuracy of embryo genome predictions.

Prediction of maternal-only or paternal-only heterozygous (het) sites is shown with and without genome sequencing of grandparents. Sites determined by comparison with Illumina trio sequencing (including the offspring) to have poor genotype quality scores or genotypes that violated Mendelian inheritance were discarded for the purpose of evaluating accuracy.

| **Embryo** | **Predicted Karyotype** | **HBA status by clinical test** | **Our predictions** | **Confirmed status** |
| --- | --- | --- | --- | --- |
| E1 | -- | -- | -- | -- |
| E2 | 46; XY | Normal | Normal | Normal |
| E3 | 45; XX, Mono 22 | -- | -- | -- |
| E4 | 46; XY | Carrier (MED) | Carrier (MED) | -- |
| E5 | -- | -- | -- | -- |
| E6 | 46; XY | Normal | Normal | -- |
| E7 | -- | -- | -- | -- |
| E8 | -- | -- | -- | -- |
| E9 | 46; XY | Carrier (MED) | Carrier (MED) | -- |
| E10 | 69; XXY | -- | -- | -- |

Table **S4: Prediction of aneuploidy across embryos and comparison with Parental Support predictions.**

Predictions obtained using SNP Array and Parental Support. For 4 embryos no DNA was amplified from the embryo biopsy which meant no predictions were made. For 2 embryos aneuploidy accounted for a lack of predictions over the chromosome. Embryo E2 was ultimately transferred into the patient. --: Not Available; MED: Mediterranean deletion of *HBA1/HBA2.*

| **Costs** | **$/family** |
| --- | --- |
| Reagents and plastic ware to amplify and prepare libraries for 288 wells | ~$2,000 |
| Sequencing reagent costs for 12 lanes of Illumina HiSeq 2000* at $1,360/lane | ~$16,000 |
| CytoSNP array costs | ~$3,000 |
| **Total** | **~$21,000** |

Table S**5:** **Estimate of costs involved in this study.**

*Paired-end 100bp sequencing reads
